# DLGAP5 promotes lung adenocarcinoma growth via upregulating PLK1 and serves as a therapeutic target

**DOI:** 10.1186/s12967-024-04910-8

**Published:** 2024-02-27

**Authors:** Maojian Chen, Shaoping Zhang, Fan Wang, Junyi He, Wei Jiang, Li Zhang

**Affiliations:** 1 Department of Medical Oncology, State Key Laboratory of Oncology in South China, Guangdong Provincial Clinical Research Center for Cancer, Sun Yat-Sen University Cancer Center, Guangzhou, 510060 Guangdong People’s Republic of China; 2 Department of Respiratory Oncology, Guangxi Medical University Cancer Hospital, Nanning, 530021 Guangxi People’s Republic of China

**Keywords:** DLGAP5, PLK1, LUAD, AT9283

## Abstract

**Background:**

Human discs large-associated protein 5 (DLGAP5) is reported to play a pivotal role in regulating the cell cycle and implicate in tumorigenesis and progression of various cancers. Our current research endeavored to explore the prognostic value, immune implication, biological function and targeting strategy of DLGAP5 in LUAD through approaches including bioinformatics, network pharmacology analysis and experimental study.

**Methods:**

Multiple databases, including TCGA, GEO, CPTAC and Human Protein Atlas, were utilized to explore the expression and clinical significance of DLGAP5 in LUAD. The genetic alterations of DLGAP5 were assessed through cBioPortal and COSMIC databases. The relationship between DLGAP5 expression and genetic abnormalities of driver genes in LUAD was analyzed through TIMER2.0 database. CancerSEA database was utilized to explore the function of DLGAP5 in 14 different states in LUAD at single-cell resolution. GDSC database was utilized to analyze the impact of DLGAP5 on IC50 of frequently-used anti-LUAD drugs. CIBERSORT method and TIMER2.0 database was utilized to explore the relationship between DLGAP5 and tumor immune infiltration. Network pharmacology was applied to screen potential DLGAP5 inhibitor. In vitro and in vivo experiments were utilized to evaluate biological function and downstream targets of DLGAP5, and the effect of screened DLGAP5 inhibitor on LUAD growth.

**Results:**

High DLGAP5 expression was commonly observed in LUAD and associated with mutation of major driver genes, poor prognosis, high IC50 values of frequently-used anti-LUAD drugs, increasing immune infiltration and elevated immune checkpoint blockade-related genes in LUAD. PLK1 was revealed as a potential DLGAP5 downstream target in LUAD. DLGAP5 overexpression or knockdown significantly promoted or inhibited LUAD cell proliferation and PLK1 expression. PLK1 overexpression well rescued DLGAP5 knockdown-induced cell proliferation inhibition, or vice versa. Furthermore, by virtual screening of an investigational drug library from the DrugBank database, AT9283 was screened and identified as a novel DLGAP5 inhibitor. AT9283 effectively suppressed growth of LUAD cells both in vitro and in vivo. DLGAP5 overexpression significantly reversed AT9283-induced proliferation inhibition. Moreover, AT9283 significantly suppressed DLGAP5 and PLK1 expression, while DLGAP5 overexpression significantly reversed AT9283-induced PLK1 suppression.

**Conclusion:**

Our research has demonstrated that DLGAP5 is upregulated in LUAD and exhibits a strong correlation with unfavorable prognosis. Furthermore, DLGAP5 assumes a significant function in the regulation of tumor immunity and treatment outcome of immune checkpoint inhibitors. Of note, we found that DLGAP5 promotes cell proliferation of LUAD via upregulating PLK1. Targeting DLGAP5 by AT9283, our newly identified DLGAP5 inhibitor, suppresses LUAD growth. DLGAP5 may become a promising prognostic biomarker and therapeutic target for patients with LUAD.

**Supplementary Information:**

The online version contains supplementary material available at 10.1186/s12967-024-04910-8.

## Introduction

Lung cancer remains the leading cause of cancer-related fatalities, making up approximately 18% of total cancer deaths globally [1]. Most lung cancer patients are diagnosed at an advanced stage, either locally or metastatic [2]. Lung adenocarcinoma (LUAD) is the most prevalent histologic subtype of lung cancer, accounting for about 50% of cases [2]. So far, despite significant improvements in the treatment strategies (e.g. chemotherapy, radiotherapy, immunotherapy and targeted therapy), the prognosis of patients with LUAD remains unsatisfactory, with an average five-year survival rate less than 20% [3]. This may due to remarkable aggressiveness and lack of effective therapeutic targets [4, 5]. Thus, identifying novel biomarkers with exceptional specificity and sensitivity is paramount for precise diagnosis, individualized treatment, and accurate prognosis prediction of LUAD.

Discs large homologous affinity protein 5 (DLGAP5), also named DLG7 or HURP, localizes within chromosome 14q22.3 [6]. DLGAP5, originally recognized as a cell-cycle-regulated protein, plays a key role in the control of M phase progression via modulating the functions of spindle assembly, kinetochore fibers (K-fibers) stabilization and chromosomal segregation during mitosis [7, 8]. In 2003, Tsou et al. firstly reported elevated DLGAP5 expression in hepatocellular carcinoma, especially during the G2/M phase [7]. DLGAP5 overexpression could maintain the cell growth in anchorage-independent and low serum-dependent manners, indicating its crucial role in oncogenic transformation [7]. Subsequently, accumulating evidences confirmed that DLGAP5, functioning as an oncogene, was frequently overexpressed in various malignancies and tightly related to poor prognosis of patients [7, 9–11]. For example, Zhang et al. [12] found that DLGAP5 was remarkably upregulated in ovarian cancer and its higher expression was correlated with poorer prognosis for survival, while the inhibition of DLGAP5 resulted in suppressed cell proliferation, G2/M phase arrest and apoptosis induction in ovarian cancer [12]. Kuo et al. [13]. reported that sorafenib, a tyrosine kinase inhibitor, could enhance the sensitivity of hepatocellular carcinoma cells to taxol through inhibition of DLGAP5 expression. Chen et al. [14]. found that bisphenol A interacted with DLGAP5 to promote proliferation, migration and invasion of osteosarcoma while silencing DLGAP5 was able to reverse the effect of bisphenol A on proliferation, migration and invasion. A recent genome-scale analysis found that DLGAP5 were highly expressed in lung cancer samples, and presented the ability to diagnose lung cancer and predict the prognosis [15], suggesting a vital role in the occurrence and development of lung cancer.

However, given the heterogeneity, the detailed roles of abnormal DLGAP5 expression in clinical significance, carcinogenic effects, tumor immunology and biological function in LUAD and the mechanisms by which DLGAP5 modulates LUAD development are currently not fully understood, which need to be further explored.

Herein, in this research, we aim to systematically explore the prognostic value, immune implication, biological function and the targeting strategy of DLGAP5 in LUAD through approaches including bioinformatics, network pharmacology analysis and experimental study.

## Materials and methods

### Data source

The transcriptome and clinical data of LUAD patients were downloaded from Cancer Genome Atlas (TCGA) database (https://cancergenome.nih.gov). And the RNA expression profiles of normal lung tissues were downloaded from Genotype Tissue Expression Project (GTEx) database (https://commonfund.nih.gov/GTex). The gene expression profiles (GSE31210, GSE43458, GSE30219, GSE32863 and GSE75073) were downloaded from Gene Expression Omnibus (GEO) database (https://www.ncbi.nlm.nih.gov/gds). In addition, several website databases that were also applied in this study would be detailly introduced below.

### Differential expression analysis

DLGAP5 mRNA expression between LUAD and normal lung tissues in TCGA, GTEx and GEO database were first compared. Then the patients with LUAD in TCGA were stratified into high- and low-DLGAP5 groups in compliance with the median score of DLGAP5 mRNA expression. |log_2_ (fold change)|> 1.5 and adjust *P*-value < 0.05 were considered as statistically significant. Moreover, clinical proteomic tumor analysis consortium (CPTAC) [16] in UALCAN [17] online database was utilized to analyze DLGAP5 protein expression level between LUAD and normal lung tissues. Human Protein Atlas (HPA) [18] online database was further applied to confirm the intensity of DLGAP5 immunohistochemical staining in LUAD. Western blot analysis was conducted to compare DLGAP5 protein expression level between normal lung epithelial cell line (BEAS-2B) and LUAD cell lines (A549, H1299, H1993, PC9, H3255, H1975).

### Genetic alteration analysis

cBioPortal [19] (https://www.cbioportal.org/) database was utilized to evaluate the alteration frequency and mutation site of DLGAP5 in LUAD. Catalogue of Somatic Mutations in Cancer (COSMIC) [20] (https://cancer.sanger.ac.uk/cosmic) database was applied to evaluate the mutation types of DLGAP5 in LUAD.

TIMER2.0 [21] (http://timer.cistrome.org/) online database was employed to investigate the relationship between DLGAP5 expression level and the genetic abnormalities of driver genes in LUAD.

### Single-cell analysis

CancerSEA [22] (http://biocc.hrbmu.edu.cn/CancerSEA/home.jsp) online database was employed to investigate the function of DLGAP5 in 14 different states in LUAD at single-cell resolution, encompassing angiogenesis, apoptosis, invasion, EMT, differentiation, proliferation, DNA damage, metastasis, hypoxia, inflammation, cell cycle, DNA repair, stemness, and quiescence.

### Functional enrichment analysis

Gene Ontology (GO) and Kyoto Encyclopedia of Genes and Genomes (KEGG) pathway analyses were performed to clarify gene enrichment difference between high- and low-DLGAP5 groups in LUAD.

### Immune infiltration analysis

The single-sample Gene Set Enrichment Analysis (ssGSEA) was realized by GSVA package [23] in R to analyze infiltration of 24 types of immune cells [24] between high- and low-DLGAP5 groups in LUAD.

### Immune checkpoint blockade (ICB)-relevant genes analysis

Knowing that expression patterns of immune checkpoint blockade (ICB)-relevant hub targets might contribute to immunotherapy efficacy [25], we compared the expression levels of the known ICB-relevant genes between high- and low-DLGAP5 groups in LUAD. Besides, TIMER2.0 [21] (http://timer.cistrome.org/) online database was employed to validate the relationship between DLGAP5 and ICB-relevant genes in LUAD.

### Drug sensitivity analysis

Genomics of Drug Sensitivity in Cancer [26] (GDSC, https://www.cancerrxgene.org/) database was applied to analyze the impact of DLGAP5 on IC50 of frequently-used therapeutic drugs for the treatment of LUAD using “pRRophetic” package in R.

### Survival analysis

The Kaplan–Meier method was employed to analyze the survival probability between high- and low-DLGAP5 groups in LUAD. Univariate and multivariate Cox regression analyses were performed to evaluate the impact of DLGAP5 expression and other clinicopathological variables on patient outcomes.

### Molecular docking and molecular dynamics simulation

The structure of the AT9283 was obtained through the PubChem website (https://pubchem.ncbi.nlm.nih.gov/). The structure of DLGAP5 protein was downloaded from the Protein Data Bank database [27] (http://www1.rcsb.org/). The main molecular docking process involved the preparation of receptor proteins and small molecule ligands, extraction and separate storage of original ligands, followed by AutoDock Vina (https://vina.scripps.edu/)-assisted molecular docking. PyMOL software was utilized to generate visualization of the resulting plots.

Furthermore, molecular dynamics (MD) simulation of 50 ns was conducted using the Gromacs2019 package (https://manual.gromacs.org) to evaluate the binding stability of the DLGAP5-AT9283 complex. The Amber14SB force field was employed for protein modeling, while the GAFF2 force field was utilized for small molecule simulations. The complex system was solvated in a water box using the TIP3P water model and electrically neutralized with an appropriate number of ions such as Na^+^ and Cl^–^. During elastic simulation, electrostatic interactions were respectively treated by the verlet and CG algorithms. The steepest descent method was employed to minimize the energy for a maximum of 50,000 steps. Both the Coulomb force cutoff distance and van der Waals radius were set at 1.4 nm. The systems were equilibrated by simulations of NVT and NPT ensembles. Then 50 ns MD simulation was performed at normal temperature and pressure. In the process of MD simulation, the LINCS algorithm was employed to constrain hydrogen bonds with an integral step of 2 fs. The V-rescale temperature coupling method was employed to regulate the simulated temperature at 300 K, while the Berendsen method was utilized to maintain the pressure at 1 bar. The root-mean-square deviation (RMSD) was utilized to monitor the allosteric changes of local sites during the simulation.

### Cell culture

Human normal lung epithelial cell line (BEAS-2B) and lung adenocarcinoma cell lines (A549, H1299, H1993, PC9, H3255, H1975) were obtained from Cell Culture Bank of Chinese Academy of Sciences (Shanghai, China) and American Type Culture Collection (ATCC; Manassas, VA, USA). All cell lines were cultured in Roswell Park Memorial Institute (RPMI) 1640 Medium (Gibco, Rockville, MD, USA) containing 10% fetal bovine serum (FBS; Gibco, Rockville, MD, USA), and 1% penicillin–streptomycin (Hyclone, Logan, UT, USA) in 5% CO_2_ atmosphere at 37 °C.

### siRNA design, plasmid construction and cell transfection

The small interfering RNA (siRNA) targeting DLGAP5 (siDLGAP5): 5′-GCAUUCCACAACAAACUACAUdTdT-3′ (sense) and 5′-AUGUAGUUUGUUGUGGAAUGCdTdT-3′ (anti-sense), negative control siRNA (siNC): 5′-UUCUCCGAACGUGUCACGUdTdT-3’(sense) and 5′-ACGUGACACGUUCGGAGAAdTdT-3′ (anti-sense) were synthesized by Guangzhou Youming Biological Technology Co., LTD (Guangzhou, China). The CDS region of human DLGAP5 or PLK1 was respectively cloned into a pcDNA3.1-HA plasmid. Cells were transfected with siRNA or plasmid using Lipofectamine™ 3000 reagent (Invitrogen; MA, USA), following the manufacturers’ instructions. The knockdown or overexpression efficiency of DLGAP5 was evaluated by western blot.

### Cell viability assay

Cells were transfected with siRNA-DLGAP5 or siRNA-negative control for 24 h prior to being seeded in 96-well plates at a density of 3 × 10^3^ cells/100μL/well. Five wells were repeated in each group. Following a 24-h incubation period for attachment, cells were administrated with specified concentrations of AT9283 (Cat No: S1134, Selleck, Houston, TX, USA) for designated durations. Subsequently, 10 μL of CCK-8 solution (Dojindo Laboratories, Kumamoto, Japan) was added to each well and incubated continuously for an additional 2 h. Finally, the absorbance value (at OD = 450 nm) was detected.

### Clone formation assay

Cells were initially transfected with siRNA-DLGAP5 or siRNA-negative control for 24 h. Thereafter, the cells (500 cells/well) were seeded in 12-well plates and cultured for 7 days to allow visible clones appeared. Followed, the cells were fixed with 4% paraformaldehyde for 15 min and then stained with 1% crystal violet for 20 min. Finally, the cells were photographed and the cell colonies (≥ 50 cells) were counted.

### Western blot analysis

Western blot analysis was conducted according to previously described methods [28]. In brief, protein lysates (20 μg/sample) from cells or tissues were separated by SDS-PAGE, transferred onto PVDF membranes, blocked with 5% non-fat milk, probed with corresponding primary antibodies and HRP-conjugated anti-rabbit IgG secondary antibody, and finally visualized using an enhanced chemiluminescence system (BioRad, Hercules, CA, USA). The antibodies used in the analysis were as follows: anti-DLGAP5 (Cat No: 12038-1-AP, 1:1000 dilution) and anti-GAPDH (Cat No: 10494-1-AP, 1:50 000 dilution) (both from Proteintech Group, Inc; Rosemont, IL, USA). anti-PLK1 (Cat No: 4513S, 1:1000 dilution) (from Cell Signaling Technology, Danvers, MA, USA). The HRP-conjugated anti-rabbit IgG secondary antibody (Cat No: W4011, 1:3000 dilution) (from Promega (Beijing) Biotech Co., Ltd; Beijing, China).

### In Vivo study

This study complies with all relevant ethical regulations. The experimental procedures involving mice were conducted in accordance with the guidelines of the Institutional Animal Care and Use Committee (IACUC) of the Sun Yat-sen University Cancer Center. Female BALB/c nude mice (4- or 5-week-old, weighing 14–16 g) were procured from Animal Research Center of Sun Yat-sen University Cancer Center and maintained under specific pathogen-free condition. H1299 cells (5 × 10^6^/200 μl) were subcutaneously injected into the right flank of each mouse. Once the tumor volume reached approximately 100 mm^3^, the mice were randomly assigned to two groups (five mice per group): Group I (Vehicle group) received equal volume of saline intraperitoneal administration as AT9283 group at the corresponding time point; Group II (AT9283 group) received an intraperitoneal injection of AT9283 (20 mg/kg) daily for 5 consecutive days, followed by a two-day interval, over a period of 3 weeks. Dosing regime of AT9283 for the experimental animals was mentioned in a previous study [29]. During the experiment, the body weight and tumor size of each mouse were measured every four days. The tumor volume (V) was estimated using the formula: V = L(length) × W (width)^2^/2. The mice were euthanized at the conclusion of the study, and their tumors were excised, photographed, weighed and harvested.

### Statistical analysis

Statistical analyses were performed using R software (Version 4.1.1) or GraphPad Prism 8 Software (San Diego, CA, USA). Measurement data were presented as means ± standard deviation (SD) and analyzed using Student's t-test for two groups or one-way analysis of variance (ANOVA) for multiple groups. Spearman correlation analysis was employed to investigate the relationship between two variables. The differences in survival between these two groups were assessed using Kaplan–Meier Survival analysis with a log-rank significance test. A *P*-value less than 0.05 was considered statistically significant.

## Results

### High DLGAP5 expression in LUAD

**Fig. 1:**
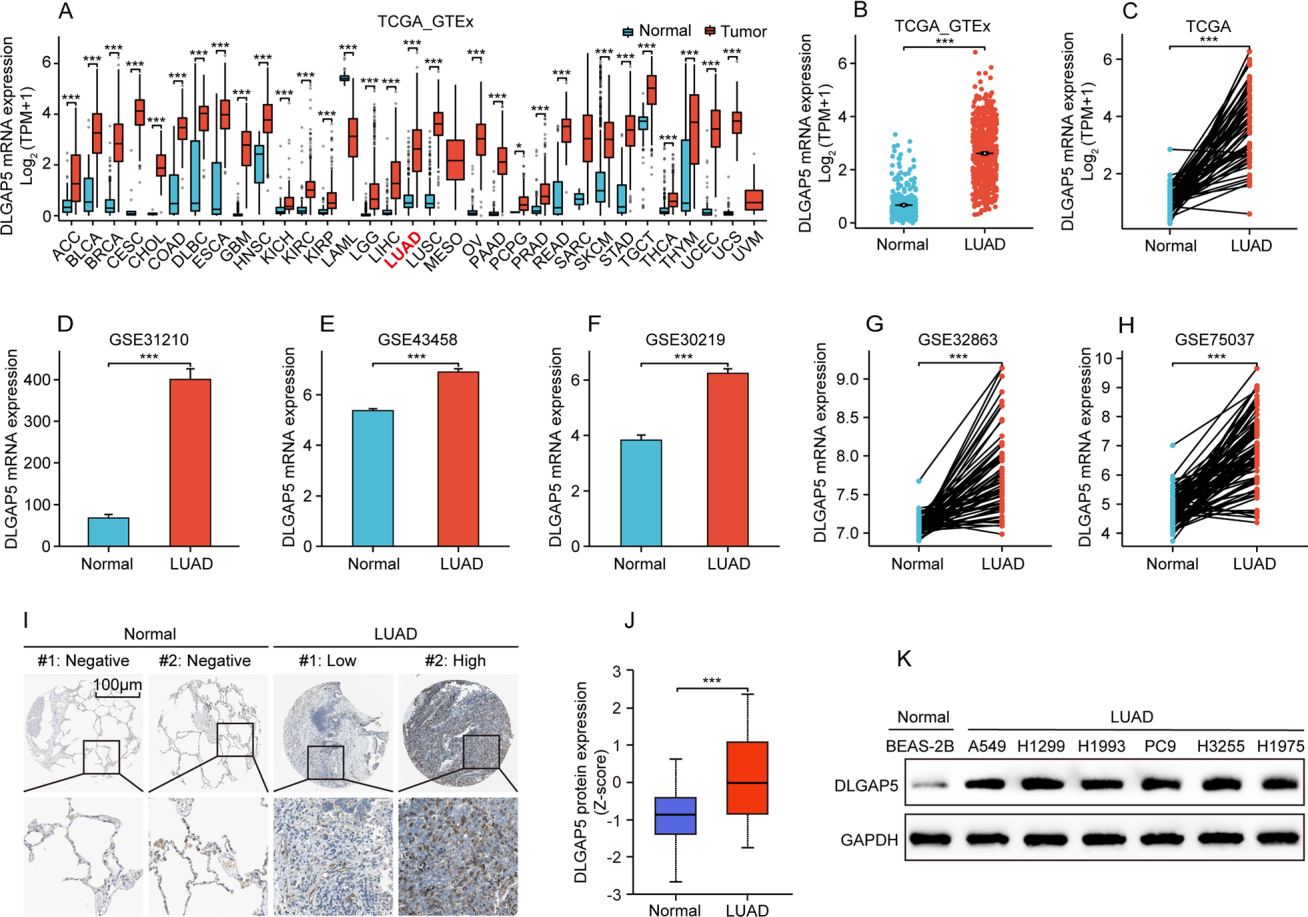
Differential expression level of DLGAP5 mRNA and protein in LUAD. **A** The comparative expression of DLGAP5 mRNA between pan-cancers including LUAD and normal tissues in TCGA & GTEx databases (^*^P < 0.05; ^***^P < 0.001). **B** Comparison of DLGAP5 mRNA expression between LUAD and normal tissues in TCGA & GTEx databases (^***^P < 0.001). **C** Comparison of DLGAP5 mRNA expression between LUAD and corresponding matched-normal tissues in TCGA database (^***^P < 0.001). **D**–**F** Comparison of DLGAP5 mRNA expression between LUAD and normal tissues in independent GEO datasets: GSE31210, GSE43458 and GSE30219 (^***^P < 0.001). **G**–**H** Comparison of DLGAP5 mRNA expression between LUAD and corresponding matched-normal tissues in independent GEO datasets: GSE32863 and GSE75037 (^***^P < 0.001). **I** Representative images of DLGAP5 protein level in LUAD detected by IHC staining in HPA database. **J** Comparison of DLGAP5 protein expression between LUAD and normal tissues in CPTAC database (^***^P < 0.001). (**K**) Western blot analysis of DLGAP5 protein expression level in LUAD cell lines (A549, H1299, H1993, PC9, H3255, H1975) and the normal lung epithelial cell line (BEAS-2B)

Firstly, we analyzed the mRNA expression level of LGAP5 in different human cancer tissues including LUAD. Data from TCGA and GTEx databases showed that DLGAP5 mRNA expression was dramatically upregulated in multiple type of cancer tissues including LUAD (Fig. 1A, B). Similar results were seen in cancer tissues and matched non-cancer tissues (Fig. 1C). In addition, high mRNA expression level of DLGAP5 in LUAD was verified in five independent GEO datasets: GSE31210, GSE43458, GSE30219, GSE32863 and GSE75037 (Fig. 1D–H).

Moreover, we further examined the protein expression level of DLGAP5 in LUAD. IHC staining data from HPA database revealed that DLGAP5 protein expression in LUAD was higher than that in adjacent tissues (Fig. 1I), which was reconfirmed by data from the CPTAC database (Fig. 1J). Besides, we conducted western blot assay to detect the protein expression level of DLGAP5 in six LUAD cell lines (A549, H1299, H1993, PC9, H3255, H1975). The results showed a significant elevation of DLGAP5 protein level in these LUAD cell lines compared to the normal lung epithelial cell line BEAS-2B (Fig. 1K). Taken together, these findings suggest that DLGAP5 may exert an oncogenic effect in the progression of LUAD.

### Genetic alterations of DLGAP5 in LUAD

**Fig. 2:**
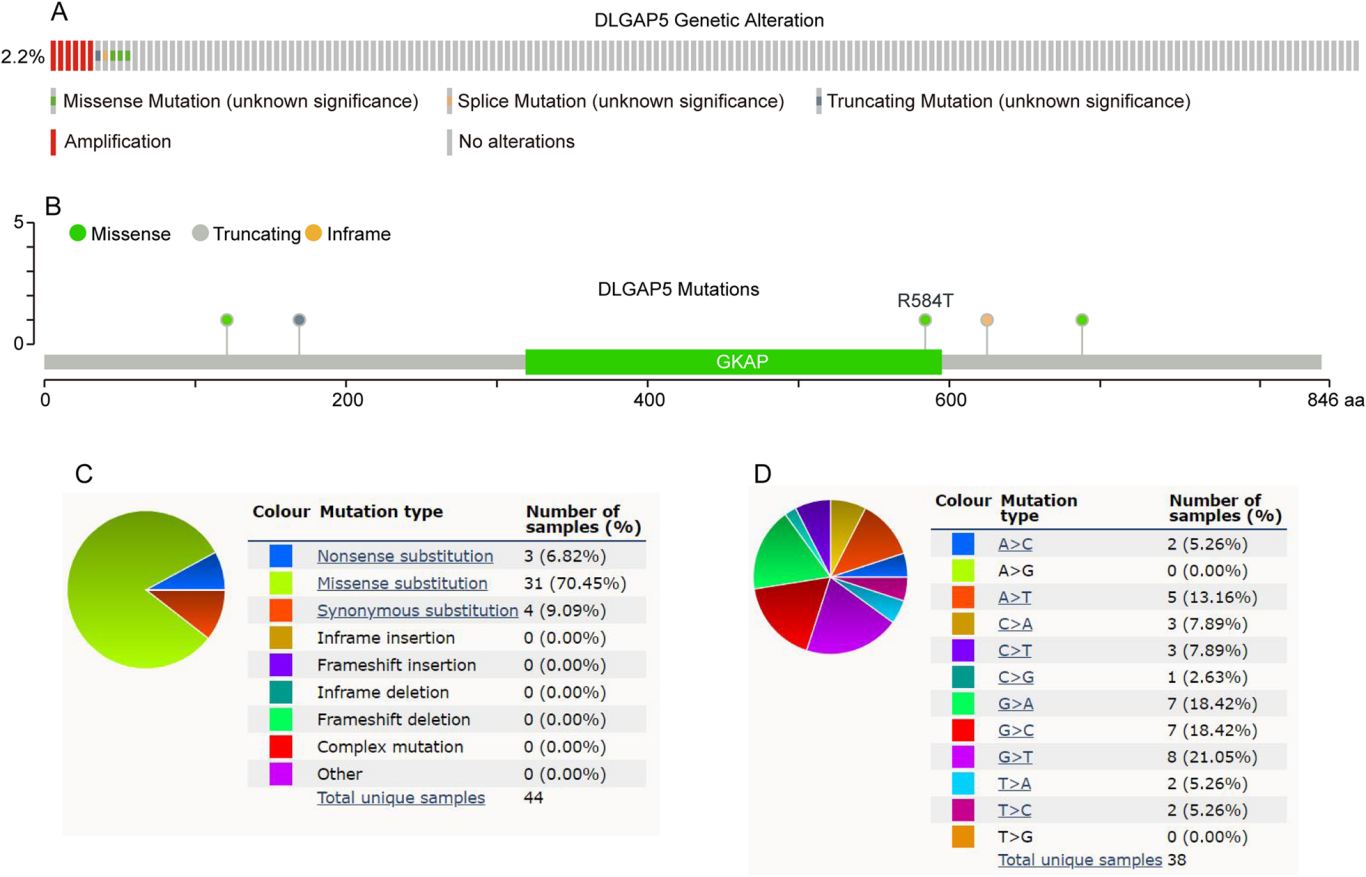
Genetic alterations of DLGAP5 in LUAD. **A** The OncoPrint of DLGAP5 alterations in LUAD in cBioPortal database. **B** Mutation diagram of DLGAP5 across protein domains in LUAD in cBioPortal database. **C** The overview of mutation types of DLGAP5 in LUAD in COSMIC database. **D** The overview of substitution mutation types of DLGAP5 in LUAD in COSMIC database

We then analyzed the alteration types and frequency of DLGAP5 via cBioPortal database using TCGA-LUAD dataset containing 507 samples with complete DNA sequencing data. The alteration frequency of DLGAP5 was 2.2% in LUAD, which included amplification in six cases, truncating mutation in one case, splice mutation in one case and missense mutation in three cases (Fig. 2A). Mutation landscapes further displayed the types, sites, and case numbers of DLGAP5 gene modification (Fig. 2B). In addition, we further assessed the mutation types of DLGAP5 in COSMIC database. As shown in the pie chart, among the 44 samples with DLGAP5 mutation, 31 had missense substitutions (70.45%), 4 had synonymous substitutions (9.09%), and 3 had nonsense substitutions (6.82%) (Fig. 2C). The substitution mutations mainly included G > T (8/38; 21.05%), G > A (7/38; 18.42%), G > C (7/38; 18.42%), A > T (5/38; 13.16%), C > A (3/38; 7.89%), C > T (3/38; 7.89%), A > C (2/38; 5.26%), T > A (2/38; 5.26%), T > C (2/38; 5.26%), and C > G (1/38; 2.63%) (Fig. 2D).

### DLGAP5 correlates with genetic abnormalities of major driver genes in LUAD

**Fig. 3:**
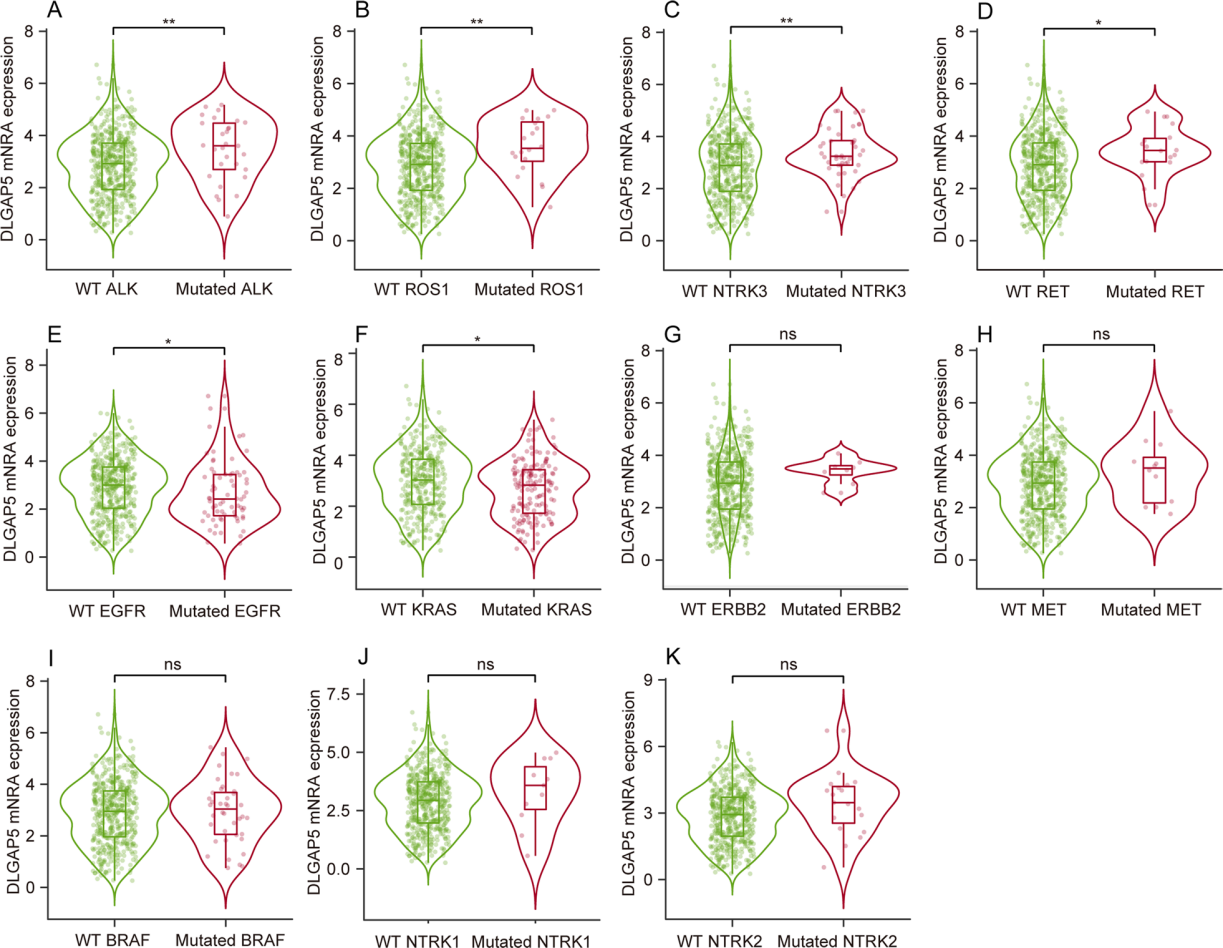
Correlation between DLGAP5 expression and genetic abnormalities of driver genes in LUAD in TIMER 2.0 database. **A**–**K** DLGAP5 expression level in wild type and mutated group of ALK (**A**), ROS1 (**B**), NTRK3 (**C**), RET (**D**), EGFR (**E**), KRAS (**F**), ERBB2 (**G**), MET (**H**), BRAF (**I**), NTRK1 (**J**), NTRK2 (**K**). (^*^P < 0.05; ^**^P < 0.01; ns, not significant)

Driver genes including EGFR, ALK, ROS1, BRAF, KRAS, MET, RET, ERBB2 and NTRK1/2/3, are recommended to detect in LUAD. Therefore, we also analyzed the correlation between DLGAP5 and those genetic abnormalities. Notably, as compared to corresponding wild type group, higher DLGAP5 expression level was found in ALK, ROS1, NTRK3 or RET mutated group (*P* < 0.05 or *P* < 0.01) (Fig. 3A–D), and lower DLGAP5 expression level in EGFR or KRAS mutated group (*P* < 0.05) (Fig. 3E–F) while DLGAP5 expression level was insignificant between the wild type and mutated group of ERBB2, MET, BRAF, NTRK1 or NTRK2 (*P* > 0.05) (Fig. 3G–K). Given that elevated DLGAP5 expression level is closely associated with the major driver genes (ALK, ROS1, NTRK3, RET) mutations in LUAD, it is reasonable to speculate that DLGAP5 may be implicated in the progression of LUAD.

### Functional states of DLGAP5 in LUAD scRNA-seq datasets

**Fig. 4:**
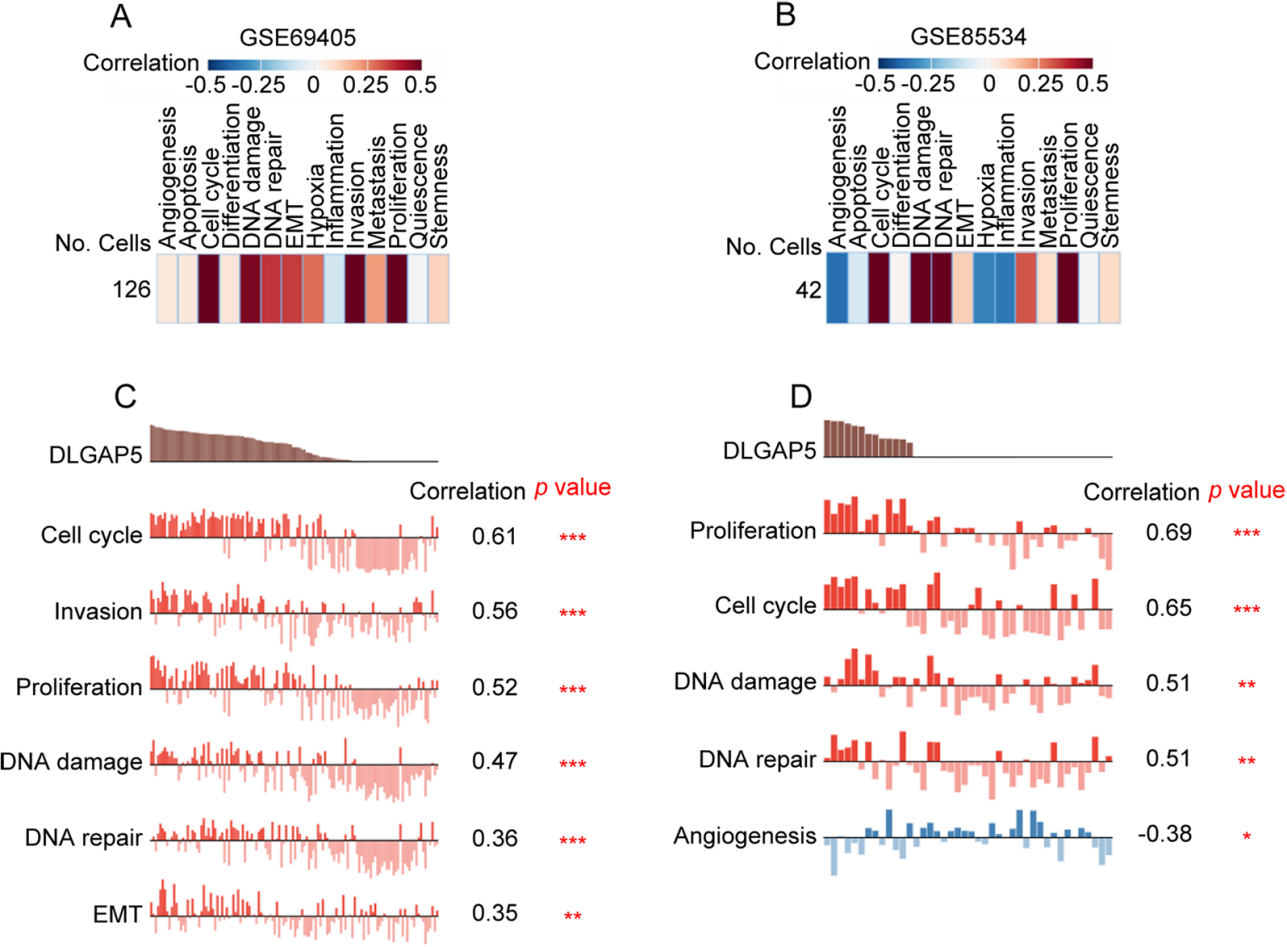
Single-cell functional analysis of DLGAP5 in LUAD using CancerSEA. **A** and **B** Correlation between DLGAP5 expression and fourteen different cancer functional states in LUAD. **C** and **D** Correlation between DLGAP5 expression and significantly different functional states in LUAD (^*^P < 0.05; ^**^P < 0.01; ^***^P < 0.001)

Next, to capture the expression of DLGAP5 at single-cell resolution and its correlation with cancer functional states in LUAD, we conducted an analysis via CancerSEA database using two GEO datasets: GSE69405 and GSE85534. Figure 4A and B showed that relationship between DLGAP5 expression and fourteen different cancer functional states in LUAD. As depicted in Fig. 4C and D, DLGAP5 expression exhibited positive correlation with functional states such as proliferation, cell cycle progression, DNA damage and DNA repair in LUAD in both datasets. The findings further suggest that DLGAP5 may play a pivotal role in the malignant process of LUAD.

### DLGAP5 correlates with immune infiltration in LUAD

**Fig. 5:**
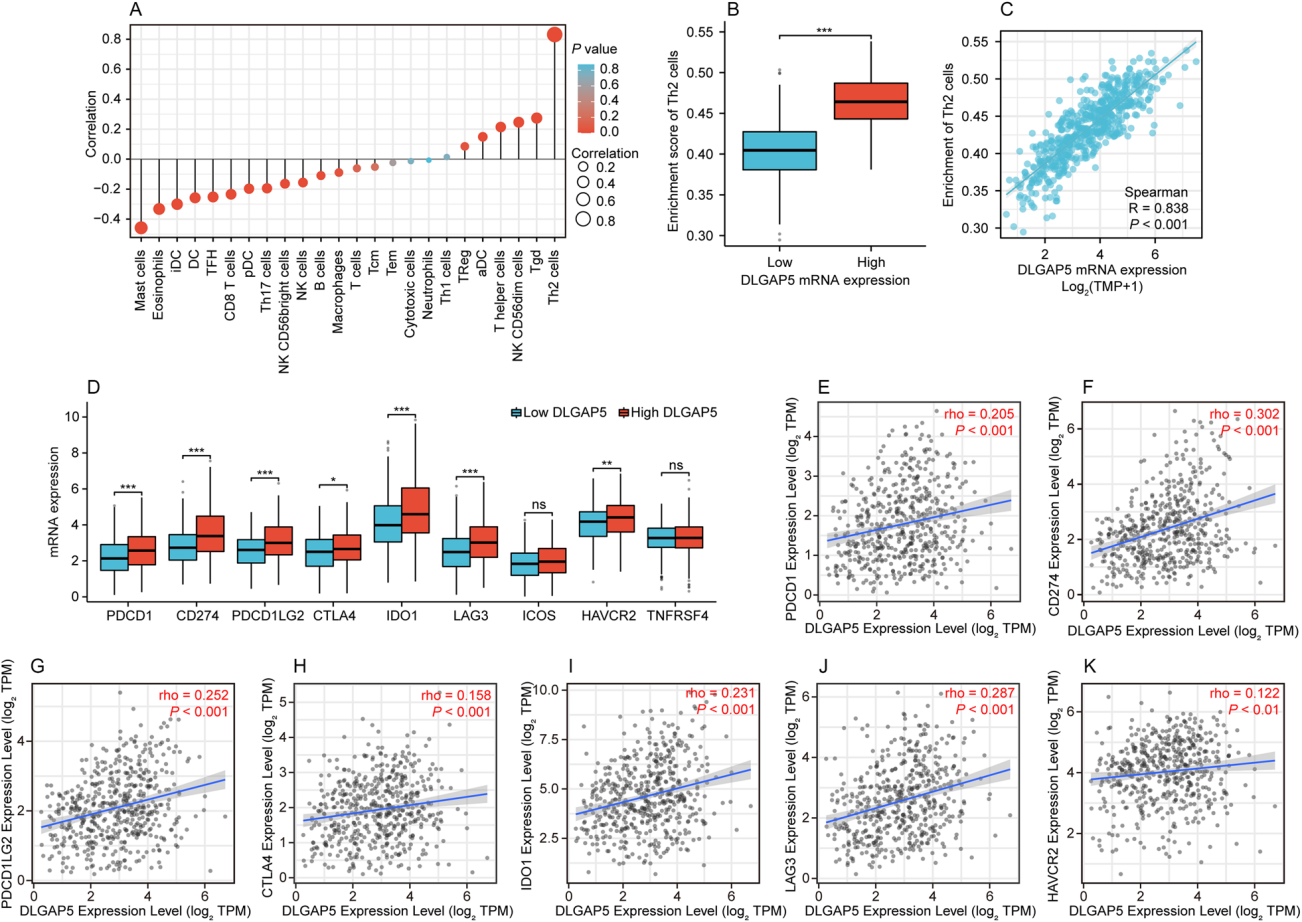
Correlations between DLGAP5 expression and immune infiltration and immune-checkpoint blockades (ICB)-relevant genes in LUAD. **A** Correlation between DLGAP5 expression and the relative abundances of 24 types of immune cells. **B** Th2 cell infiltration level in low- and high-DLGAP5 expression groups (^***^P < 0.001). **C** Correlation between DLGAP5 expression and Th2 cell infiltration. **D** Differences in mRNA expression of ICB-relevant genes (PDCD1, CD274, PDCD1LG2, CTLA4, IDO1, LAG3, ICOS, HAVCR2 and TNFRSF4) between high- and low-DLGAP5 groups in TCGA-LUAD cohort (^*^P < 0.05; ^**^P < 0.01; ^***^P < 0.001; ns, not significant). **E**–**K** Correlation between DLGAP5 expression and ICB-relevant genes of PDCD1 (**E**), CD274 (**F**), PDCD1LG2 (**G**), CTLA-4 (**H**), IDO1 (**I**), LAG3 (**J**) and HAVCR2 (**K**) in TIMER 2.0 database

To further investigate the possible mechanism by which elevated DLGAP5 expression promotes the malignant process of LUAD, we evaluated the relationship between DLGAP5 and immune cell infiltration since tumor microenvironment is closely related to tumor development. We categorized the data from LUAD patients into high- and low-DLGAP5 expression groups for comparative analysis. As shown in Fig. 5A, DLGAP5 expression were closely correlated with immune cell subsets, including Th2 cells, Tgd, NK CD56dim cells, T helper cells, aDC, B cells, NK CD56bright cells, pDC, CD8 T cells, DC, NK cells, TFH, iDC, Eosinophils, Th17 cells and Mast cells (*P* < 0.05). Notably, DLGAP5 expression had especially positive correlation with the level of Th2 cell infiltration (R = 0.838, *P* < 0.001) (Fig. 5B, C). These findings reveal that DLGAP5 is closely involved in immune infiltration in LUAD.

### DLGAP5 correlates with ICB-relevant genes in LUAD

Previous research has confirmed that multiple ICB-relevant genes may coordinately influence the local tumor-immune environment, thereby determining the efficacy of immunotherapy [25]. Based on these, we wonder whether DLGAP5 associates with the vital ICB-relevant genes and involves in the regulation of tumor microenvironment. As shown in Fig. 5D, among the nine known ICB vital targets, seven genes were found to exhibit higher expression level in high-DLGAP5 group compared to low-DLGAP5 group, namely PDCD1, CD274, PDCD1LG2, CTLA4, IDO1, LAG3 and HAVCR2. In addition, the analysis from TIMER2.0 database showed that DLGAP5 expression positively correlated with the seven ICB-relevant genes (Fig. 5E–K). Although these correlations were weak/moderate, they were statistically significant. These findings may imply a pivotal role of DLGAP5 in the tumor-immune microenvironment of LUAD.

### DLGAP5 correlates with drug sensitivity in LUAD

**Fig. 6:**
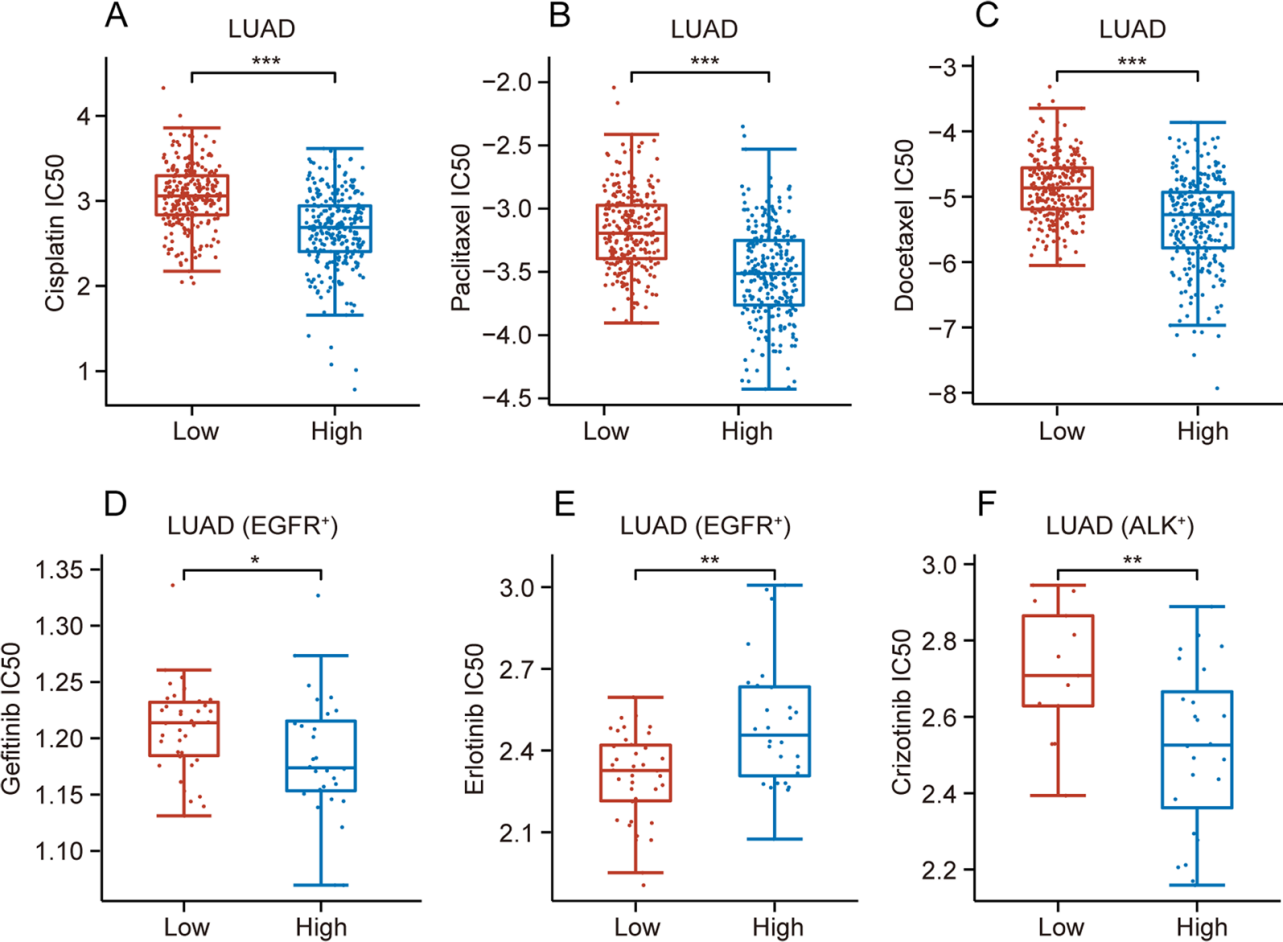
The results of analysis between DLGAP5 expression and diverse drug sensitivity. **A**–**C** IC50 values of frequently-used chemotherapeutic drugs (cisplatin(**A**), paclitaxel (**B**) and docetaxel (**C**) in high- and low-DLGAP5 groups in LUAD (^***^P < 0.001). **D**–**E**) IC50 values of frequently-used molecular targeted therapeutic drugs (gefitinib (**D**) and erlotinib (**E**) in high- and low-DLGAP5 groups in EGFR-mutant (EGFR^+^) LUAD (^*^P < 0.05; ^**^P < 0.01). **F** IC50 value of frequently-used molecular targeted therapeutic drug crizotinib in high- and low-DLGAP5 groups in ALK-mutant (ALK^+^) LUAD (^**^P < 0.01)

To further explore the impact of DLGAP5 expression on the sensitivity of frequently-used anti-LUAD drugs, we compared their IC50 values between high- and low-DLGAP5 groups. As illustrated in Fig. 6A–D and F, we found that the IC50 values of several anti-LUAD drugs decreased in the high-DLGAP5 group, including chemotherapeutic drugs (cisplatin, paclitaxel, docetaxel), EGFR inhibitor (gefitinib) and receptor tyrosine kinase (RTK) inhibitor (crizotinib), revealing that patients with high DLGAP5 expression were relatively sensitive to these anti-cancer drugs. In contrast, the IC50 value of erlotinib (an EGFR inhibitor) increased in high-DLGAP5 group in EGFR-mutant (EGFR^+^) LUAD (Fig. 6E). These results suggest that EGFR-mutant LUAD patients with high DLGAP5 expression may confer resistance to erlotinib treatment.

### Correlation between DLGAP5 and clinicopathologic factors in LUAD

**Fig. 7:**
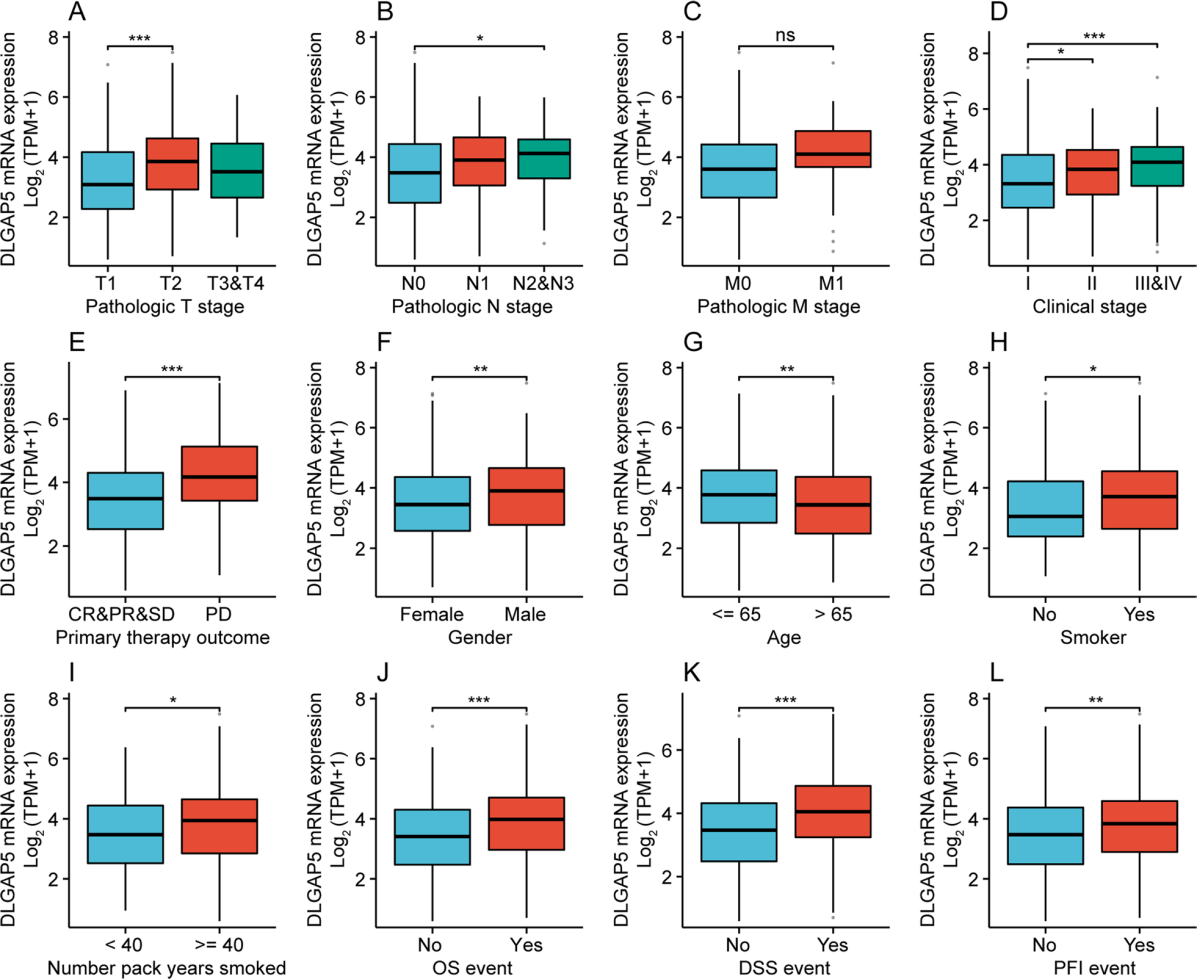
Correlation between DLGAP5 expression and different clinicopathologic characteristics of LUAD. **A**–**L** Correlation between DLGAP5 expression and T stage (**A**), N stage (**B**), M stage (**C**), clinical stage (**D**), primary therapy outcome (**E**), gender (**F**), age (**G**), smoker (**H**), number_pack_years_smoked (**I**), OS event (**J**), DSS event (**K**) and PFI event (**L**). (^*^P < 0.05; ^**^P < 0.01; ^***^P < 0.001; ns, not significant)

We next explored the possible relationships between DLGAP5 and multiple clinicopathologic factors. As seen in Table 1 and Fig. 7A–L, the expression of DLGAP5 was found to be significantly correlated with T stage (T1 *vs.* T2, *P* < 0.001), N stage (N0 *vs.* N2&N3, *P* < 0.05), clinical stage (stage I *vs.* stage III&IV, *P* < 0.001), primary therapy outcome (CR&PR&SD *vs.* PD, *P* < 0.001), gender (female *vs.* male, *P* < 0.01), age (≤ 65 *vs.* > 65, *P* < 0.01), smoker (no *vs.* yes, *P* < 0.05), number_pack_years_smoked (< 40 *vs.* ≥ 40, *P* < 0.05), OS event (no *vs.* yes, *P* < 0.001), DSS event (no *vs.* yes, *P* < 0.001) and PFI event (no *vs.* yes, *P* < 0.01). The findings suggest that DLGAP5 expression correlates with aggressive behavior and poor treatment outcome in LUAD.

**Table 1:** Clinicopathologic factors of high- and low-DLGAP5 expression groups in TCGA-LUAD cohort

Characteristics	Low expression of DLGAP5	High expression of DLGAP5	*P* value
n	269	270	
Pathologic T stage, n (%)			0.002
T1	107 (20%)	69 (12.9%)	
T2	126 (23.5%)	166 (31%)	
T3	26 (4.9%)	23 (4.3%)	
T4	8 (1.5%)	11 (2.1%)	
Pathologic N stage, n (%)			< 0.001
N0	192 (36.7%)	158 (30.2%)	
N1	39 (7.5%)	58 (11.1%)	
N2	25 (4.8%)	49 (9.4%)	
N3	0 (0%)	2 (0.4%)	
Pathologic M stage, n (%)			0.084
M0	182 (46.7%)	183 (46.9%)	
M1	8 (2.1%)	17 (4.4%)	
Clinical stage, n (%)			0.001
Stage I	168 (31.6%)	128 (24.1%)	
Stage II	57 (10.7%)	68 (12.8%)	
Stage III	30 (5.6%)	54 (10.2%)	
Stage IV	9 (1.7%)	17 (3.2%)	
Primary therapy outcome, n (%)			< 0.001
PD	20 (4.5%)	51 (11.4%)	
SD	23 (5.1%)	15 (3.3%)	
PR	4 (0.9%)	2 (0.4%)	
CR	181 (40.3%)	153 (34.1%)	
Gender, n (%)			0.002
Female	162 (30.1%)	127 (23.6%)	
Male	107 (19.9%)	143 (26.5%)	
Race, n (%)			0.350
Asian	6 (1.3%)	2 (0.4%)	
Black or African American	30 (6.4%)	25 (5.3%)	
White	207 (43.9%)	202 (42.8%)	
Age, n (%)			0.023
< = 65	117 (22.5%)	140 (26.9%)	
> 65	146 (28.1%)	117 (22.5%)	
Smoker, n (%)			0.020
No	48 (9.1%)	29 (5.5%)	
Yes	215 (41%)	233 (44.4%)	
Number pack years smoked, n (%)			0.010
< 40	102 (27.6%)	86 (23.3%)	
> = 40	74 (20.1%)	107 (29%)	
OS event, n (%)			< 0.001
No	194 (36%)	153 (28.4%)	
Yes	75 (13.9%)	117 (21.7%)	
DSS event, n (%)			< 0.001
No	209 (41.6%)	174 (34.6%)	
Yes	44 (8.7%)	76 (15.1%)	
PFI event, n (%)			0.001
No	175 (32.5%)	138 (25.6%)	
Yes	94 (17.4%)	132 (24.5%)	

PD, Progressive Disease; SD, stable disease; PR, partial response; CR, complete response; OS, overall survival; DSS, disease-specific survival; PFI, progression-free interval

### Prognostic value of DLGAP5 in LUAD

**Fig. 8:**
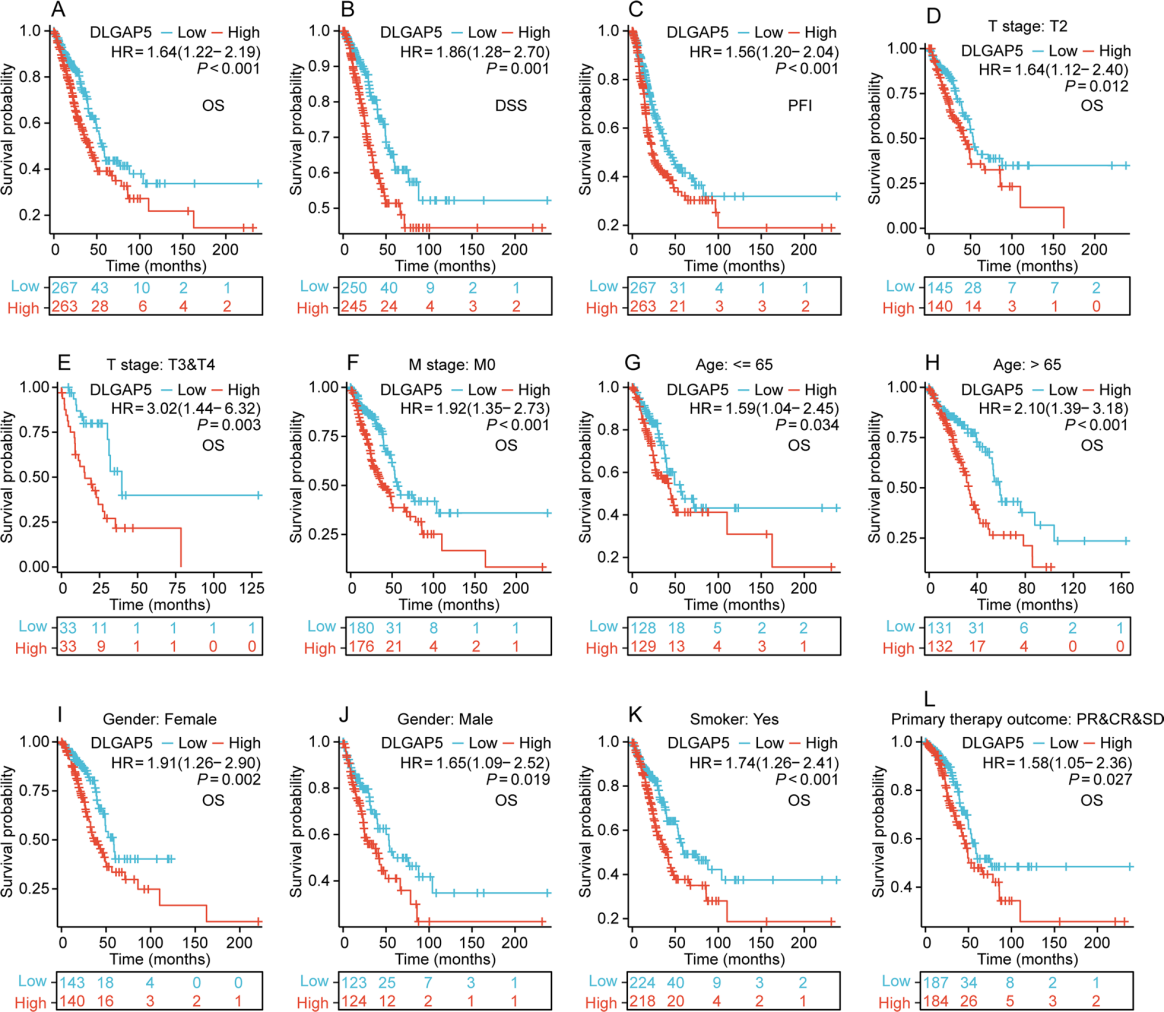
Prognostic value of DLGAP5 expression in patients with LUAD. **A**–**C** The prognostic value of DLGAP5 expression in OS (**A**), DSS (**B**) and PFI (**C**) of all patients with LUAD. **D**–**L** The prognostic value of DLGAP5 expression in different subgroups, including T2 stage (**D**), T3&T4 stage (**E**), M0 stage (**F**), Age ≤ 65 (**G**), Age > 65 (**H**), Gender-female (**I**), Gender-male (**J**), Smoker (**K**) and Primary therapy outcome-(PR&CR&SD) (**L**)

Followed, we sought to identify whether DLGAP5 expression affects the prognosis of LUAD patients. As expected, we found that the high-DLGAP5 group displayed a significantly poorer prognosis than the low-DLGAP5 group, as evidenced by shorter overall survival (OS), disease-specific survival (DSS), and progression-free interval (PFI) (all *P* < 0.01) (Fig. 8A–C). Further subgroup analysis showed that patients with high DLGAP5 expression had shorter OS in subgroups labeled as T2 stage, T3&T4 stage, M0 stage, age ≤ 65 years, age > 60 years, female, male, smoker and primary therapy outcome (PR&CR&SD) (all *P* < 0.05) (Fig. 8D–L). Moreover, univariate Cox regression analysis showed that TNM stage, clinical stage, tumor status, primary therapy outcome, and high expression of DLGAP5 were associated with poor OS (*P* < 0.05). Meanwhile, further multivariate Cox regression analysis revealed that high DLGAP5 expression was an independent prognostic factor for OS (HR = 1.615, 95% CI 1.065–2.448, *P* < 0.05) (Table 2). Altogether, these findings imply that high DLGAP5 expression is associated with an unfavorable prognosis in patients diagnosed with LUAD.

**Table 2:** Univariate and multivariate Cox regression analyses of DLGAP5 expression for overall survival (OS) in TCGA-LUAD cohort

Characteristics	Total (n)	Univariate analysis		Multivariate analysis	
		Hazard ratio (95% CI)	*P* value	Hazard ratio (95% CI)	*P* value
Pathologic T stage	527		< 0.001		
T1	176	Reference		Reference	
T2	285	1.507 (1.059–2.146)	0.023	1.033 (0.619–1.726)	0.900
T3&T4	66	3.095 (1.967–4.868)	< 0.001	1.681 (0.778–3.635)	0.187
Pathologic N stage	514		< 0.001		
N0	345	Reference		Reference	
N1	96	2.293 (1.632–3.221)	< 0.001	1.644 (0.740–3.653)	0.222
N2&N3	73	2.993 (2.057–4.354)	< 0.001	2.221 (0.780–6.323)	0.135
Pathologic M stage	381		0.010		
M0	356	Reference		Reference	
M1	25	2.176 (1.272–3.722)	0.005	1.482 (0.535–4.105)	0.449
Clinical stage	522		< 0.001		
Stage I	292	Reference		Reference	
Stage II	123	2.341 (1.638–3.346)	< 0.001	0.959 (0.414–2.224)	0.923
Stage III&Stage IV	107	3.635 (2.574–5.132)	< 0.001	1.090 (0.334–3.564)	0.886
Primary therapy outcome	442		< 0.001		
CR&PR&SD	371	Reference		Reference	
PD	71	3.673 (2.578–5.234)	< 0.001	4.284 (2.730–6.720)	< 0.001
Gender	530		0.570		
Female	283	Reference			
Male	247	1.087 (0.816–1.448)	0.569		
Age	520		0.185		
< = 65	257	Reference			
> 65	263	1.216 (0.910–1.625)	0.186		
Smoker	516		0.777		
No	74	Reference			
Yes	442	0.942 (0.625–1.420)	0.775		
DLGAP5	530		< 0.001		
Low	267	Reference		Reference	
High	263	1.638 (1.224–2.192)	< 0.001	1.615 (1.065–2.448)	0.024

PD, Progressive Disease; SD, stable disease; PR, partial response; CR, complete response

### DLGAP5 is required for LUAD Cell proliferation

**Fig. 9:**
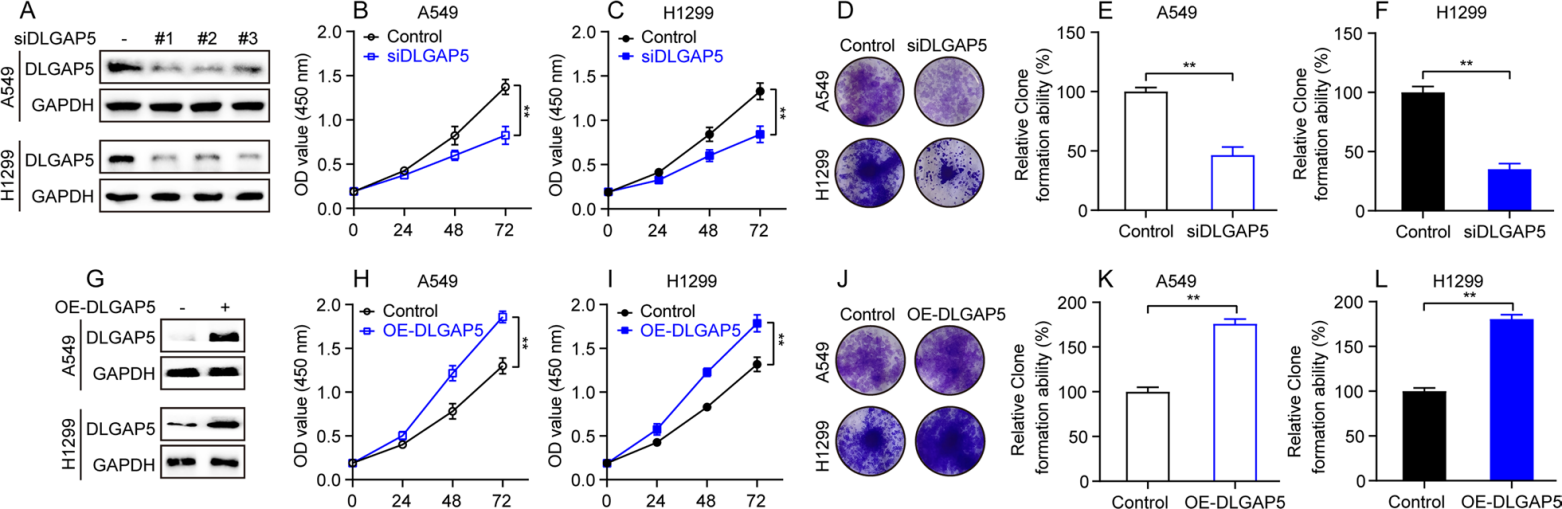
DLGAP5 is required for LUAD cell proliferation. **A** Cells were transfected with siRNA against DLGAP5 and collected 48 h later for evaluation of DLGAP5 knockdown efficiency by western blot assay. **B**–**C** Cells transfected with siDLGAP5 were subjected to CCK-8 assay. Data are presented as mean ± SD, ^**^P < 0.01. **D** Cells transfected with siDLGAP5 were subjected to clone formation assay. **E**–**F** Quantitative analysis of **D** results and data are presented as mean ± SD, ^**^P < 0.01. **G** Cells were transfected with DLGAP5-overexpression plasmid and collected 48 h later for evaluation of DLGAP5 overexpression efficiency by western blot assay. **H**–**I** Cells transfected with DLGAP5-overexpression plasmid were subjected to CCK-8 assay. Data are presented as mean ± SD, ^**^P < 0.01. **J** Cells transfected with DLGAP5-overexpression plasmid were subjected to clone formation assay. **K**–**L** Quantitative analysis of (**J**) results and data are presented as mean ± SD, ^**^P < 0.01

The preceding bioinformatic results revealed that DLGAP5 may play an important role in the pathogenesis and progression of LUAD, which needed to be further verified by experiments, so we evaluated the impact of DLGAP5 on LUAD cell proliferation in vitro. siRNA targeting DLGAP5 was transfected into A549 and H1299 cells, resulting in a significant reduction of DLGAP5 protein levels as confirmed by western blot analysis, indicating successful knockdown efficiency (Fig. 9A). CCK-8 and clone formation assays showed that DLGAP5 knockdown displayed a remarkable anti-proliferative effect on A549 and H1299 cells (Fig. 1B–F). In order to further verify the role of DLGAP5 on LUAD cell proliferation, pcDNA3.1 plasmid harboring CDS region of DLGAP5 was transfected into A549 and H1299 cells, resulting in a remarkable upregulation of DLGAP5 protein level as evidenced by western blot analysis, indicating successful overexpression efficiency (Fig. 9G). As anticipated, CCK-8 and clone formation assays showed that DLGAP5 overexpression remarkedly accelerated proliferation of A549 and H1299 cells (Fig. 9H–L). Altogether, these findings suggest that DLGAP5 is required for LUAD cell proliferation.

### DLGAP5 promotes LUAD cell proliferation via upregulating PLK1

**Fig. 10:**
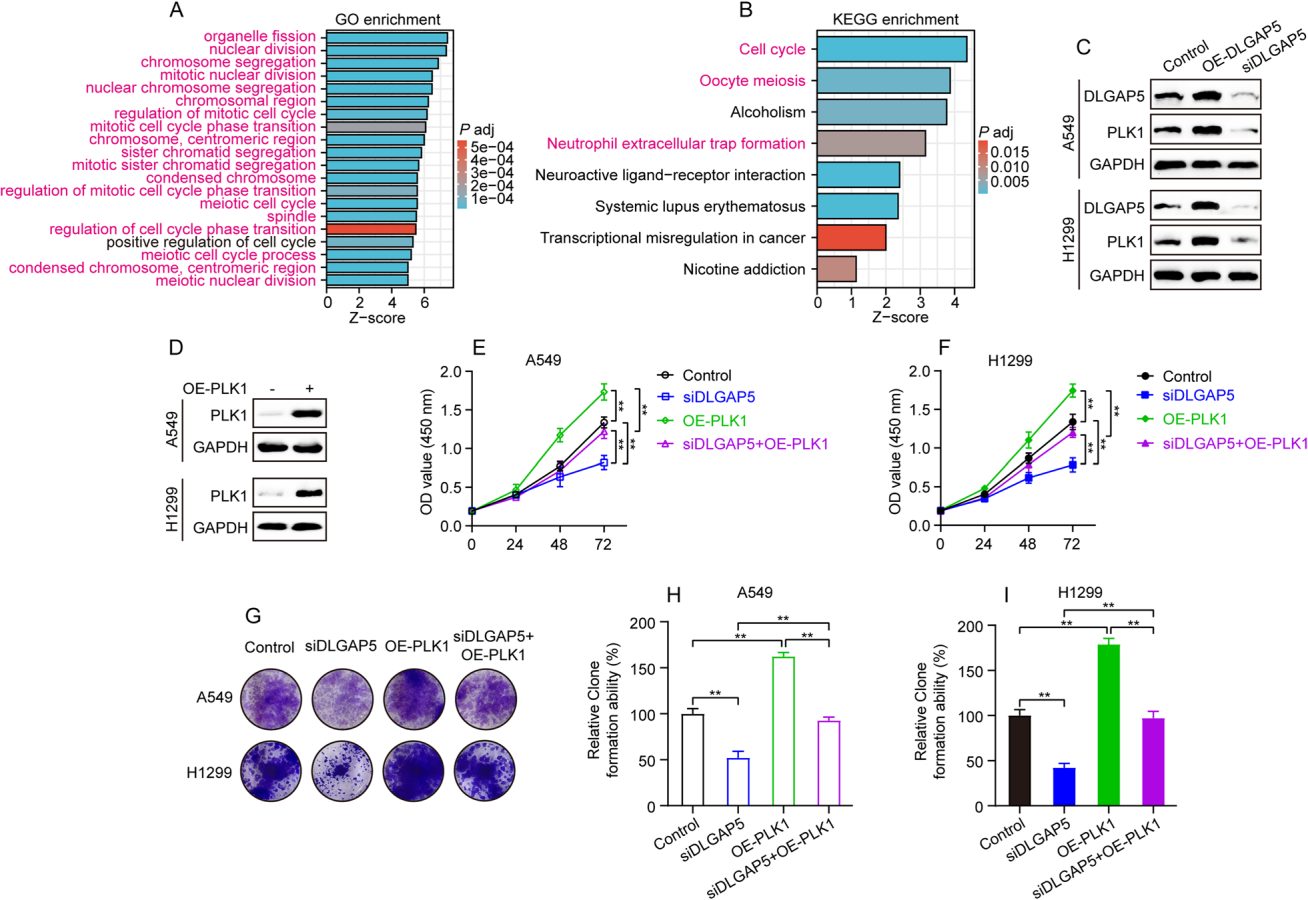
DLGAP5 promotes LUAD cell proliferation via upregulating PLK1. **A** GO terms analysis of DLGAP5-related differentially expressed genes (DEGs), shown are top 20 GO terms, color of magenta marked GO terms including PLK1 gene. **B** KEGG analysis of DLGAP5-related DEGs, color of magenta marked KEGG terms including PLK1 gene. **C** Cells were transfected with DLGAP5-overexpression plasmid or siRNA against DLGAP5, and collected 48 h later for detection of DLGAP5 and PLK1 by western blot assay. **D** Cells were transfected with PLK1-overexpression plasmids and collected 48 h later for evaluation of PLK1 overexpression efficiency by western blot assay. **E**–**F** Cells transfected with either siDLGAP5 or PLK1-overexpression plasmid, or both were subjected to CCK-8 assay. Data are presented as mean ± SD, ^**^P < 0.01. **G** Cells transfected with either siDLGAP5 or PLK1-overexpression plasmid, or both were subjected to clone formation assay. **H**–**I** Quantitative analysis of **G** results and data are presented as mean ± SD, ^**^P < 0.01

We next sought to investigate the underlying mechanism by which DLGAP5 promotes LUAD cell proliferation. According to the cutoff criteria (|log_2_FC|< 1.5 and adjusted *P* < 0.05), we totally identified 781 differentially expressed genes (DEGs) between high- and low-DLGAP5 expression groups from TCGA-LUAD cohort, consisting of 538 upregulated genes and 243 downregulated genes. Then, both GO and KEGG analyses revealed that these DLGAP5-related DEGs were predominantly enriched in the regulation of mitotic cell cycle (Fig. 10A and B). Noteworthily, among these top 20 GO terms, PLK1 gene was found to emerge in 19 terms (19/20; 95%) (Fig. 10A and Additional file 1: Table S1). Besides, cell cycle, the top one pathway ranked in KEGG terms, was also found to contain PLK1 gene (Fig. 10B and Additional file 1: Table S2). Studies have demonstrated that PLK1 is an important regulator to coordinate cell cycle progression in rapidly proliferating cells and accelerate the occurrence and progression of multiple cancers [30, 31]. Along these lines, we wondered whether PLK1 is a key target of DLGAP5 to promote LUAD cell proliferation. Hence, the protein level of PLK1 and cell viability were measured. As expected, DLGAP5 overexpression remarkedly upregulated PLK1 in A549 and H1299 cells (Fig. 10C). On the contrary, DLGAP5 knockdown remarkedly downregulated PLK1 (Fig. 10C). Further, we successfully constructed the PLK1-overexpression plasmid and transfected it into A549 and H1299 cells (Fig. 10D). The results obtained from CCK-8 and clone formation assay showed that PLK1 overexpression could not only significantly promote proliferation of A549 and H1299 cells, but also well rescue DLGAP5 knockdown-induced cell proliferation inhibition (Fig. 10E–I). Taken together, these results suggest that DLGAP5 promotes LUAD cell proliferation via upregulating PLK1.

### AT9283 suppresses proliferation of LUAD cells via inhibiting DLGAP5/PLK1 axis

**Fig. 11:**
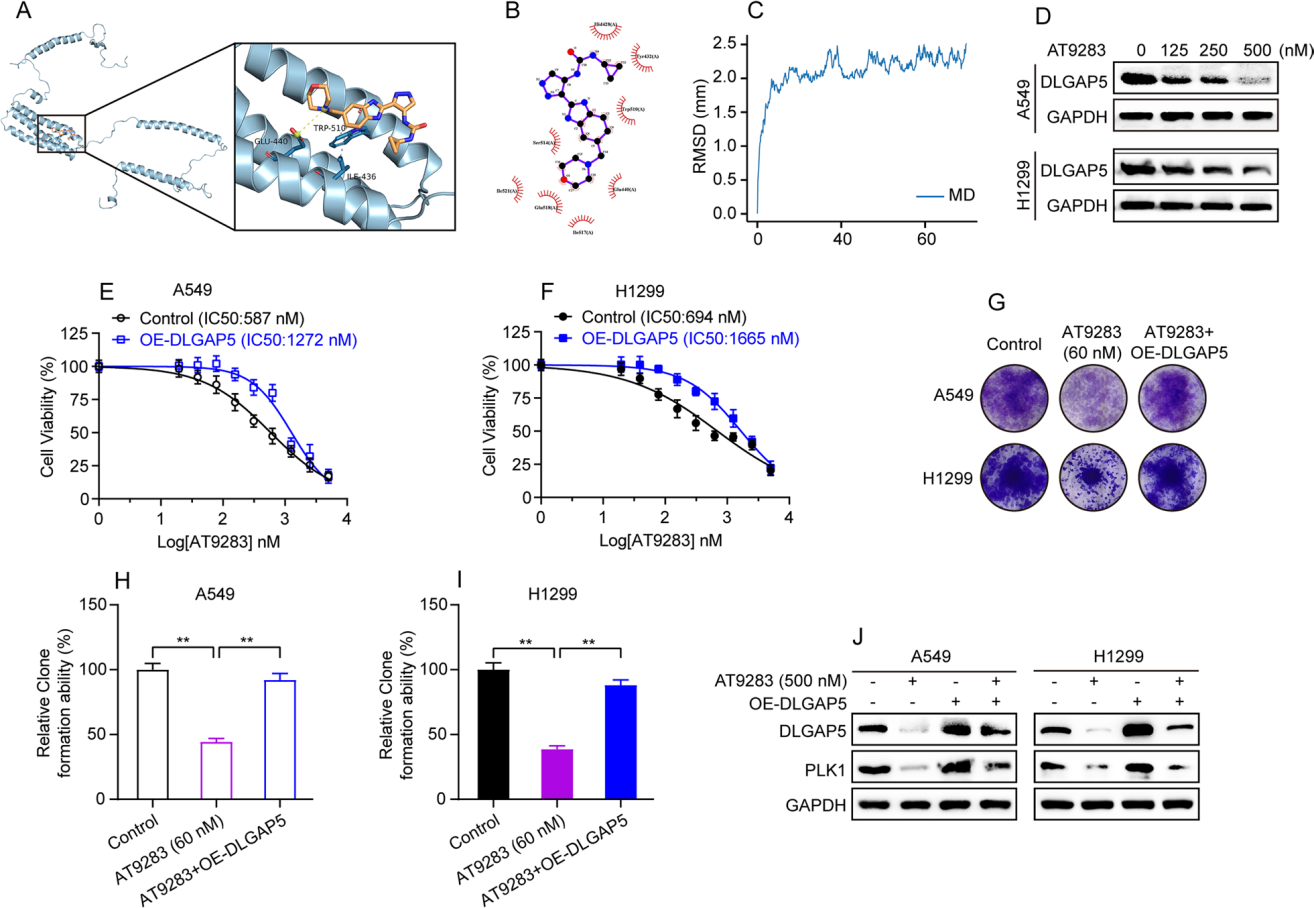
AT9283 suppresses proliferation of LUAD cells via inhibiting DLGAP5/PLK1 axis. **A** The 3D docking diagram of the interaction between DLGAP5 protein and AT9283. **B** The 2D docking diagram of the interaction between DLGAP5 protein and AT9283. **C** RMSD values for AT9283 during a 50 ns molecular dynamics simulation. **D** Cells treated with AT9283 (0 nM, 125 nM, 250 nM, 500 nM) and collected 48 h later for detection of DLGAP5 by western blot assay. **E**–**F** Cells were treated with AT9283 alone or along with DLGAP5-overexpression plasmids for 48 h. The IC_50_ of AT9283 was detected by CCK-8 assay. **G** Cells transfected with AT9283 alone or along with DLGAP5-overexpression plasmid were subjected to clone formation assay. **H**–**I** Quantitative analysis of **G** results and data are presented as mean ± SD, ^**^P < 0.01. **J** Cells transfected with AT9283 alone or along with DLGAP5-overexpression plasmid were collected 48 h later for detection of DLGAP5 and PLK1 by western blot assay

The above-mentioned findings emphasize the significant impact of DLGAP5 on LUAD cell proliferation, we reasoned that drugs targeting DLGAP5 could become a promising therapeutic strategy for LUAD patients. To preclinically prove this concept, we aimed to identify inhibitors of DLGAP5. Excitingly, by virtual screening of an investigational drug library from the DrugBank database [32], we found that AT9283 [33], a multikinase inhibitor, may be closely correlated with DLGAP5 expression. We then did molecular docking analysis and the obtained results showed low docking energy of AT9283-DLGAP5 complex (-6.8 kcal/mol) (Fig. 11A and B). Further molecular dynamics simulation of 50 ns showed that the RMSD of AT9283 was relatively stable (Fig. 11C). These results, to some extent, suggested that AT9283 may be a potential small molecule that could inhibit DLGAP5 expression. In order to verify whether AT9283 could exactly inhibit the expression of DLGAP5 in LUAD, AT9283 (0 nM, 125 nM, 250 nM, 500 nM) was administered to A549 and H1299 cells for 48 h. As expected, the western blot analysis showed that AT9283 potently inhibited the protein level of DLGAP5 in both A549 and H1299 cells in a dose-dependent manner (Fig. 11D).

The above results prompted us to further evaluate the impact of AT9283-induced DLGAP5 inhibition on LUAD cell proliferation. To this end, A549 and H1299 cells were treated with indicated concentrations of AT9283 or along with DLGAP5 overexpression plasmid. As expected, CCK-8 result showed that AT9283 significantly inhibited proliferation of both A549 (IC50: 587 nM) and H1299 (IC50: 694 nM) cells. Noteworthily, we found that DLGAP5 overexpression significantly abrogated AT9283-induced cell proliferation suppression as evidenced by an approximate two-fold increase of IC50 (Fig. 11E, F). Moreover, clone formation assay also showed that DLGAP5 overexpression was noted to significantly reverse AT9283-induced clone formation ability reduction (Fig. 11G–I). Additionally, further western blot analysis displayed that AT9283 significantly suppressed the protein level of DLGAP5 and PLK1 in A549 and H1299 cells, while DLGAP5 overexpression significantly reversed AT9283-induced PLK1 protein suppression (Fig. 11J).

Taken together, the above resulting data suggest that AT9283 suppresses proliferation of LUAD cells via inhibiting DLGAP5/PLK1 axis.

### AT9283 suppresses the tumor growth and DLGAP5/PLK1 axis in a murine LUAD xenograft model

**Fig. 12:**
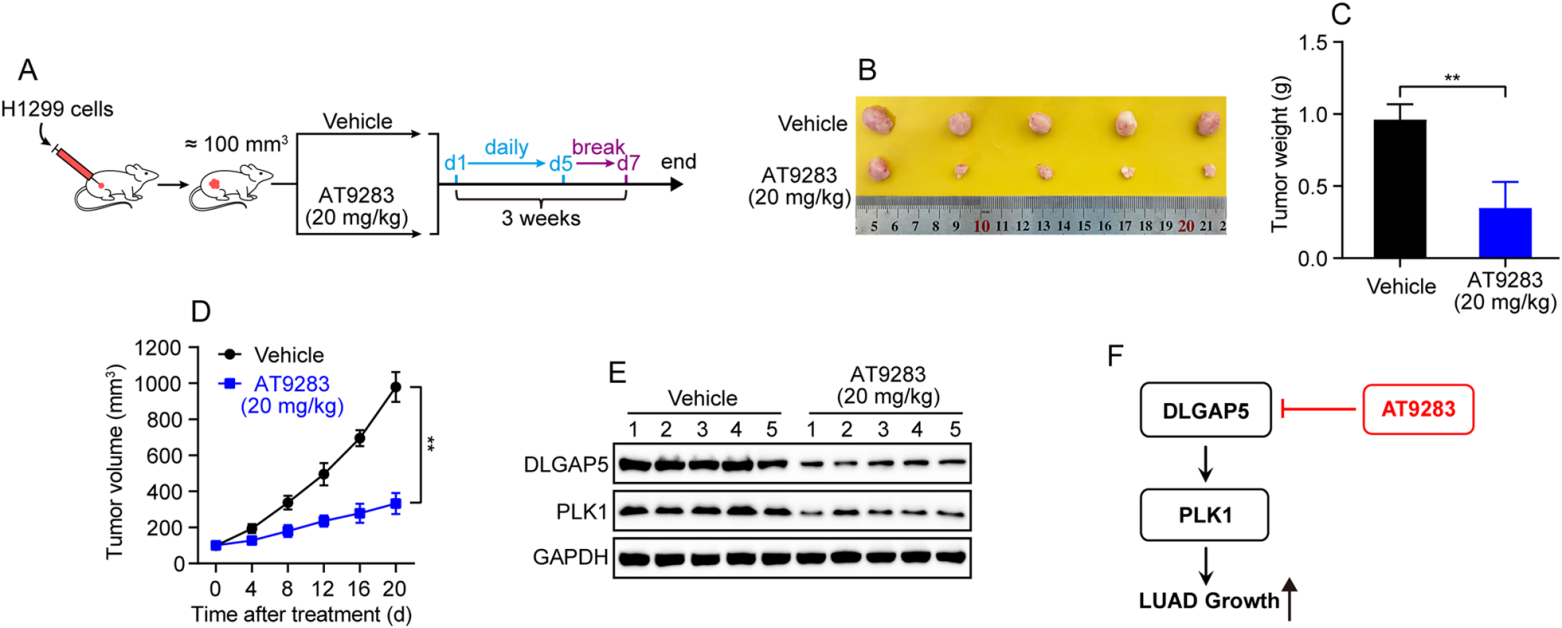
AT9283 suppresses the tumor growth and DLGAP5/PLK1 axis in a murine LUAD xenograft model. **A** Schematic illustration of the in vivo experimental design. Female BALB/c nude mice (n = 5) bearing H1299 cells were intraperitoneally treated with AT9283 (20 mg/kg) daily for 5 days followed by a two-day break for a period of 3 weeks, then tumors were harvested for analysis. **B** Photos of the formed tumors at the end of experiments. **C** Tumor weight at the end of experiments. Data are presented as mean ± SD, ^**^P < 0.01. **D** Tumor growth curve. Data are presented as mean ± SD, ^**^P < 0.01. **E** The formed tumors were collected for detection of DLGAP5 and PLK1 by western blot assay. **F** Schematic diagram of possible action mechanism by which AT9283 plays a role in LUAD growth

At last, to further assess the effect of AT9283 in vivo, a murine LUAD xenograft model of BALB/c nude mice bearing H1299 cells was established and AT9283 was administrated (Fig. 12A). As depicted in Fig. 12B–D, the AT9283 treated group exhibited a significant inhibition of tumor volume and tumor weight as compared to the vehicle treated group. Notably, further western blot analysis displayed that AT9283 treatment also led to a remarkable reduction of DLGAP5 and PLK1 protein levels in tumors (Fig. 12E). These results were consistent with the findings of in vitro experiments (Fig. 11), and jointly verified that AT9283 can attenuate LUAD growth by suppressing DLGAP5/PLK1 axis in vivo.

## Discussion

There is evidence that DLGAP5 contributes to tumorigenesis and progression of numerous cancer types, for example, bladder cancer [34], endometrial cancer [35], ovarian cancer [36] and lung cancer [37, 38]. In our current study, we conducted bioinformatics, network pharmacology analysis and experimental study to gain a more comprehensive understanding of the potential functions and regulatory mechanisms of DLGAP5 in LUAD. Initially, by bioinformatic analyses, we discovered that both transcription and translation levels of DLGAP5 were significantly elevated in LUAD. Higher DLGAP5 expression level was also found in the mutated group of major driver genes including ALK, ROS1, NTRK3 and RET in LUAD. Besides, single-cell analysis from scRNA-seq datasets uncovered that DLGAP5 was associated with various functions including proliferation, cell cycle regulation, and DNA damage response in LUAD. Moreover, high DLGAP5 was associated with the sensitivity of frequently-used anti-LUAD drugs, advanced tumor stage and poor prognosis. In vitro, we also exhibited that DLGAP5 overexpression promoted cell proliferation of LUAD, while DLGAP5 knockdown showed the opposite effect. These findings were similar with previous reports [11, 38], jointly supporting that DLGAP5 may promote LUAD development and be a promising prognostic biomarker for LUAD.

Accumulating researches have demonstrated the significant involvement of infiltrating immune cells within the tumor microenvironment in tumorigenesis and progression, thereby impacting the prognostic outcomes of patients with cancer [24, 39, 40]. Then, given that DLGAP5 promoting tumor progression in LUAD, we also investigate the relationship of DLGAP5 with immune infiltration. Our results revealed that DLGAP5 was significantly associated with various immune cells, particularly positively correlated with Th2 cells. Generally, Th2 cell is a subset of helper T cells that can induce the polarization of M2 macrophages with immunosuppressive properties, leading to the formation of suppressive tumor microenvironment and promoting tumor development [41, 42]. In view of the imbalance of Th1/Th2 contributing to LUAD development [43, 44], DLGAP5 may regulate the ratio of Th1/Th2 to promote tumor growth in LUAD. In recent years, immunotherapies, especially immune checkpoint blockade therapy, have arisen as an auspicious approach to treat various cancers, including LUAD [45, 46]. Interestingly, in our present study, we revealed another crucial aspect of the regulatory impact of DLGAP5 on tumor microenvironment, as evidenced by the positive correlations between DLGAP5 and the essential immune checkpoint blockade (ICB)-related genes in LUAD. Although these correlations are weak/moderate, they are statistically significant. Collectively, these resulting data suggest that DLGAP5 is involved in the regulation of tumor immunity to promote tumor development and may serve as a promising biomarker for immune checkpoint blockade therapy in LUAD.

Although the oncogenic role of DLGAP5 in LUAD has been evidenced, the exact regulative mechanism is incompletely understood. Studies have confirmed that PLK1, a kind of serine/threonine-protein kinase, is overexpressed and play an oncogenic role in various types of cancer [30, 47]. Noteworthily, in our study, we found that PLK1 emerged in 19 terms among the top 20 GO terms enriched from analysis of DEGs between high- and low-DLGAP5 expression groups from TCGA-LUAD cohort. Moreover, PLK1 overexpression was found to trigger proliferation of LUAD cells, confirming its oncogenic role in LUAD. We further found that DLGAP5 overexpression remarkedly upregulated PLK1 in LUAD cells, or vice versa. Besides, overexpression of PLK1 partially abrogated siDLGAP5-induced proliferation suppression. It has been reported that PLK1 overexpression is positively associated with multiple defects in cell cycle including mitosis, cytokinesis, supernumerary centrosomes, compromised cell-cycle checkpoints [48]. Ning et al. found that knockdown of PLK1 significantly impeded the transition of LUAD cells from G0/G1 to S phase [49]. Considering that PLK1 is involved in cell cycle progression, and DLGAP5 could positively regulate PLK1 expression, it is not unexpected that DLGAP5 could promote LUAD cell proliferation through cell-cycle modulation. There is evidence also supports that knockdown of DLGAP5 causes cell cycle arrest in the G1/S stage and inhibits proliferation of LUAD cells [50]. Taken together, our results, for the first time, revealed that DLGAP5 promotes LUAD development through upregulating its downstream target PLK1 and DLGAP5 might be a promising target for LUAD treatment. Whether such mechanism exists in other cancers is worth further study.

Our findings prompted us to screen effective inhibitors of DLGAP5 against LUAD. Fortunately, through network pharmacology analyses, AT9283 was identified as a potential inhibitor of DLGAP5. AT9283, originally identified as a multi-target kinase inhibitor against aurora A, Aurora B, JAK3, JAK2, and Abl, has been reported to be effective drug against leukemia cells, myeloproliferative diseases and various solid cancer cell lines [29, 33, 51, 52]. In this study, we demonstrated that AT9283 also had anti-cancer activity of LUAD in vitro and in vivo. Mechanically, AT9283 suppressed the protein level of DLGAP5 and PLK1, and DLGAP5 overexpression abrogated the AT9283-induced cell proliferation suppression and PLK1 protein downregulation in LUAD cells. Together, our findings provide cogent evidence that AT9283 may be a promising candidate targeted DLGAP5/PLK1 axis for LUAD treatment. However, the specific mechanisms through which AT9283 inhibits the expression of DLGAP5 are yet to be fully addressed. Nevertheless, there are studies reported that aurora A modulated the cell transforming activities of DLGAP5 through phosphorylating it [53, 54], and AT9283 served as a potent inhibitor targeting aurora A [29, 51]. It would be of interest to examine whether AT9283 inhibits DLGAP5/PLK1 also through targeting aurora A in LUAD.

## Conclusion

Our research has demonstrated that DLGAP5 is upregulated in LUAD and exhibits a strong correlation with unfavorable prognosis. Furthermore, DLGAP5 exerts a significant function in the regulation of tumor immunity and treatment outcome of immune checkpoint inhibitors. Of note, we found that DLGAP5 promotes cell proliferation of LUAD via upregulating PLK1. Targeting DLGAP5 by AT9283, our newly identified DLGAP5 inhibitor, suppresses LUAD growth. DLGAP5 may become a promising prognostic biomarker and therapeutic target for patients with LUAD.

### Supplementary Information

**Additional file 1: Table S1.** Gene Ontology enrichment analysis for DLGAP5-related DEGs. **Table S2.** KEGG enrichment analysis for DLGAP5-related DEGs.

## Data Availability

Data are available in a public, open access repository. Data are available upon reasonable request. All data relevant to the study are included in the article or uploaded as supplementary information.
